# SCORE2 Outperforms Pol-SCORE in Detecting Increased Cardiovascular Risk

**DOI:** 10.3390/pathophysiology32030045

**Published:** 2025-09-09

**Authors:** Magdalena Zawadzka, Ewelina Ejchman-Pac, Amelia Kowalska, Paweł Szymański, Justyna Marszałkowska-Jakubik

**Affiliations:** 1 Department of Health Education, Prevention, and Promotion, Military Institute of Hygiene and Epidemiology, Kozielska Street 4, 01-163 Warsaw, Poland; ewelina.e.pac@wihe.pl (E.E.-P.); justyna.jakubik@wihe.pl (J.M.-J.); 2 Department of Epidemiology and Public Health, Medical University of Lodz, Żeligowskiego Street 7/9, 90-752 Lodz, Poland; amelia.kowalska@student.umed.lodz.pl; 3 Department of Radiobiology and Radiation Protection, Military Institute of Hygiene and Epidemiology, Kozielska Street 4, 01-163 Warsaw, Poland; pawel.szymanski@wihe.pl; 4 Department of Pharmaceutical Chemistry, Drug Analysis and Radiopharmacy, Medical University of Lodz, Muszyńskiego Street 1, 90-151 Lodz, Poland

**Keywords:** cardiovascular disease, SCORE2, Pol-SCORE, military personnel, hypertension, obesity, tobacco use, preventive medicine, public health

## Abstract

**Background:** Cardiovascular disease (CVD) remains the leading cause of death in Europe. Despite medical advancements, modifiable risk factors—such as obesity, smoking, and physical inactivity—continue to rise, especially in high-demand professional groups like military personnel. The updated SCORE2 model offers broader assessment capabilities compared to the traditional Pol-SCORE system used in Poland. This study aimed to assess and compare cardiovascular risk using both models and evaluate self-awareness of cardiovascular risk factors among military and civilian employees. **Methods:** The study included military personnel and civilian defense employees who completed a health-related questionnaire and underwent clinical evaluation, including blood pressure measurement and lipid profiling. Cardiovascular risk was assessed using both Pol-SCORE (fatal events only) and SCORE2 (fatal and non-fatal events). Statistical analysis was conducted using standard parametric and nonparametric methods. **Results:** SCORE2 classified significantly more individuals into high or very high cardiovascular risk categories than Pol-SCORE. Differences were especially pronounced among women and civilians. Elevated blood pressure, overweight, obesity, tobacco use, and stress were commonly observed. Despite a high level of awareness about prevention, regular participation in screening was low, and many respondents underestimated their health risk, indicating the presence of unrecognized or underestimated risk. **Conclusions:** SCORE2 proves to be a more sensitive and comprehensive tool for cardiovascular risk evaluation. The findings emphasize the urgent need for targeted prevention strategies and health education, especially in high-risk occupational groups such as military personnel.

## 1. Introduction

Cardiovascular diseases (CVDs) remain the leading cause of death worldwide, contributing significantly to morbidity and mortality among working-age populations. Identifying individuals at increased cardiovascular risk is critical, particularly in professional groups exposed to chronic stress, such as military personnel [1,2,3].

Cardiovascular risk is the chance of developing or dying from heart disease over a defined period. In practice, only selected key factors can be assessed, as it is not feasible to account for all potential variables. Many risk factors are difficult to measure, costly to assess, or time-consuming—some are not yet fully understood. For this reason, the concept of general cardiovascular risk is used in daily medical practice, based on the analysis of selected, well-documented factors. Modifiable factors include, among others, smoking, insufficient intake of fruits and vegetables, excessive alcohol consumption, and lack of regular physical activity. On the other hand, hypertension, obesity, abnormal lipid profiles (including elevated LDL cholesterol and lipoprotein(a)), and type 2 diabetes—being diet-related diseases—are classified as modifiable to some extent. In addition, other emerging determinants such as high-sensitivity C-reactive protein, homocysteine, and environmental exposures like air pollution and climate-related stressors may also play an important role. These factors should be given particular attention when studying specific populations, such as soldiers, who are often exposed to extreme weather and environmental challenges [2,4]. Although soldiers are often perceived as being in good physical condition, research indicates a high prevalence of cardiovascular risk factors in this group. For instance, the Military Systematic Coronary Risk Evaluation (MIL-SCORE) program revealed that 60% of Polish soldiers had elevated LDL cholesterol levels, and nearly 45% had hypertension. Nevertheless, studies comparing cardiovascular risk classification in military personnel using validated tools such as the Polish version of the Systematic Coronary Risk Evaluation (Pol-SCORE) and its updated European counterpart SCORE2 remain scarce [1]. Therefore, effective cardiovascular disease prevention should be based on interventions that can be implemented by everyone, regardless of non-modifiable factors such as age, sex, or place of residence. Such an approach can significantly contribute to reducing premature heart attacks [3].

Although some statistical data from recent years indicate clear progress in combating heart disease and strokes, other data related to risk factors reveal alarming upward trends. For instance, Poland has achieved measurable success in reducing the percentage of smokers in recent decades. In the 1980s, 65–75% of men smoked tobacco products, while by 2010, this figure had dropped to approximately 30% [5]. In a cross-sectional study conducted in March 2022 by the National Opinion Research Panel, 30.8% of Polish men and 27.1% of women reported regular (daily) smoking. Nevertheless, in 2019 in Poland, smoking was the leading cause of death among men (26.6%) and the second leading cause among women (13.8%) [6].

It is also concerning that only 24.2% of adults meet the national physical activity recommendations [7]. Interestingly, the increase in cardiovascular diseases is by no means a new issue—it was already observed between 1922 and 1931, despite preventive efforts (an increase from 2602 in 1922 to 3234 in 1931 per 10,000 soldiers of the Polish Army) [8].

A study published in 2020 found that despite the decline in smoking prevalence, tobacco use remains the strongest behavioral risk factor for death. Arterial hypertension, on the other hand, turned out to be a stronger predictor of stroke than heart attack. Diabetes, non-HDL cholesterol, and current smoking were more significant risk factors for myocardial infarction than for stroke. Overall, modifiable risk factors accounted for approximately 70% of cardiovascular disease cases in middle-income countries, including Poland, with arterial hypertension having the greatest impact [3]. Polish research from 2023 confirmed that the prevalence of cardiovascular disease (CVD) risk factors was higher among uniformed service employees than in the general population [9].

Despite advances in diagnosis and therapy, cardiovascular diseases (CVDs) remain the leading cause of death in Europe. A particular challenge is the increasing prevalence of modifiable risk factors such as obesity, undiagnosed hypertension, and metabolic disorders [7].

The new European guidelines incorporate the latest research data to simplify recommendations regarding specific blood pressure reduction targets (Table 1) [10,11].

A healthy lifestyle remains a key element of prevention, with particular emphasis on smoking prevention [3]. It is an effective method of primary prevention of chronic diseases and mortality, as well as a means of supporting healthy aging and longevity. Considering the controllability of many of these factors, it is essential to effectively identify them and implement broad health education efforts. A useful tool that supports prevention is the Systematic Coronary Risk Evaluation (SCORE) model, published in 2003, which was developed to estimate the 10-year risk of fatal cardiovascular disease in Europe. SCORE was quickly accepted and recommended by the European Society of Cardiology for identifying risk factors and assessing cardiovascular risk [12,13]. In 2021, the model was updated to SCORE2, incorporating more recent epidemiological data and eliminating key limitations of the original model, including the underestimation of total cardiovascular risk due to the exclusion of non-fatal events [14]. In Poland, the Pol-SCORE charts, calibrated for the Polish population, have been in use since 2007 and were updated in 2015. They are based on national epidemiological data, account for demographic and mortality trends specific to Poland, and estimate 10-year cardiovascular mortality risk in adults aged 40–70 years using variables such as age, sex, smoking status, systolic blood pressure, and total cholesterol [15].

The 2021 European Guidelines on Cardiovascular Disease Prevention (SCORE2) estimate the probability of fatal and non-fatal cardiovascular events over the next 10 years in apparently healthy individuals (i.e., those without diagnosed atherosclerotic cardiovascular disease, diabetes, chronic kidney disease, or extremely high cholesterol levels). The SCORE2 risk chart is applicable to individuals aged 40 to 69 years (SCORE2-OP is a modification for individuals over 70 years of age) [16].

Military service involves high demands in terms of both physical fitness and mental resilience. It encompasses not only combat operations but also peacetime duties, such as the protection of critical infrastructure, participation in peacekeeping missions, and humanitarian operations. Soldiers must demonstrate above-average physical performance, a high level of discipline, and the ability to function under intense and prolonged stress. Despite these predispositions, they are not free from the risk of developing various diseases, including cardiovascular diseases. Moreover, the specific nature of military service—including exposure to stress, irregular lifestyle, and frequent participation in foreign missions—may contribute to the intensification of cardiovascular risk factors. Consequently, systematic health monitoring of this occupational group takes on particular importance.

## 2. Materials and Methods

### 2.1. Study Design and Population

The study population was selected in accordance with the internal procedures of the government institution. Convenience sampling was used in this study. The research was conducted in a total of seven military units. The detailed criteria for selecting the sample, due to applicable data protection regulations, remain undisclosed. All participants were informed of the details of the study, its purpose, and the benefits of participation. Each person expressed written informed consent to participate in the study and completed an original questionnaire assessing the soldier’s/military employee’s knowledge of cardiovascular diseases and risk factors for their occurrence. The survey consisted of a total of 53 questions divided into 7 thematic sections. It was conducted in the presence of qualified personnel between October 2023 and February 2024 (Supplementary File S1).

Inclusion Criteria: soldier or civilian employee of the military; provision of informed consent to participate in this study.

Exclusion Criteria: lack of informed consent to participate in this study; any clinical condition that prevents cooperation; cancer or acute chronic inflammatory conditions.

### 2.2. Cardiovascular Risk Assessment Tools

The Pol-SCORE (Systematic Coronary Risk Evaluation) scale, calibrated for the Polish population, was used to assess the overall 10-year risk of cardiovascular death, and the SCORE2 scale, calibrated for the population at high risk of cardiovascular disease, was used to assess the 10-year risk (fatal and non-fatal) of a cardiovascular event. For this purpose, blood pressure and lipid profile were measured for each participant. For Pol-SCORE, the risk was determined based on age, sex, smoking, systolic blood pressure, and total cholesterol concentration, and for SCORE2, age, sex, smoking, systolic blood pressure, and non-HDL cholesterol concentration (the difference between total cholesterol and HDL cholesterol concentration) were used. Four risk classes were distinguished in Pol-SCORE, i.e., very high: ≥10%; high: ≥5% and <10%; moderate: ≥1% and <5%, and low: <1%. SCORE 2 distinguishes three risk classes, i.e., very high—for people < 50 years of age ≥ 7.5%, respectively; for people 50–69 years of age ≥ 10%, respectively, high—for people < 50 years of age 2.5–7.5%, respectively; for people 50–69 years of age 5–10%, respectively, and low—for people < 50 years of age < 2.5%, respectively; for people 50–69 years of age < 5%.

### 2.3. Clinical and Biochemical Measurements

Blood pressure was measured by qualified medical personnel using an OMRON M7 Intelli IT device (Omron Healthcare Co., Kyoto, Japan). A single measurement was performed in a sitting position after a 5 min rest.

Blood samples were collected from 196 soldiers and civilian military personnel in Polish military units between October 2023 and February 2024. The collected blood samples were stored at 2–8 °C and sent to the laboratory for lipid profile testing.

### 2.4. Study Objectives

The aim of the study was to compare cardiovascular risk assessment using two tools: Pol-SCORE and SCORE2, in order to identify differences between them and to demonstrate why SCORE2 can be considered a more accurate tool than Pol-SCORE.

### 2.5. Statistical Analysis

The collected material was analyzed statistically using Excel tools (Version 2507 Build 16.0.19029.20136) and Statistica 13.3. Descriptive statistics and parametric and nonparametric data analysis methods were used. The assessment of normality distribution was performed using the Shapiro–Wilk test. The Mann–Whitney U test was used to assess the relationship between quantitative variables in the absence of normal distribution. In terms of the Kruskall–Wallis H test and post hoc analysis, multiple comparisons of mean ranks for all samples were used to analyze more than two groups of an ordinal variable. The assessment of the relationship between two qualitative variables was performed using the Pearson Chi^2^ test of independence. The correlation coefficient was calculated to express the interdependence between two variables. The r-Pearson correlation coefficient was used to describe the correlation between quantitative variables, and the Spearman rank correlation coefficient was used in the absence of normality distribution. For all analyses, the significance level was assumed to be *p* ≤ 0.05.

### 2.6. Ethical Considerations

This study was conducted in accordance with the Declaration of Helsinki and approved by the Institutional Review Board of the Bioethics Committee at the Military Medical Chamber in Warsaw (protocol code 11/23, approval date: 19 May 2023).

## 3. Results

### Characteristics of the Respondents

The study group consisted of 196 people, including 153 soldiers (78.1%) and 43 civilian military employees (21.9%). Almost three-quarters were men (71.4%). The respondents were aged 40–67 ($\bar{x}$ = 47.83 ± 5.32; Q1 = 44.00 and Q3 = 51.00). A total of 58.7% of people had higher education, 38.3% had secondary education, and the rest had vocational education.

According to Pol-SCORE, two-thirds of the people (66.84%) had a 10-year risk of death from cardiovascular causes described as moderate (1–5%), every fifth person (21.43%) as high (5–10%), 8.16% as low (<1%), and 3.57% as very high (>10%).

According to SCORE2, almost the same number of people had a 10-year risk of cardiovascular death described as low (for people < 50 years old, <2.5%; for people aged 50–69 years old, <5%) and high (for people < 50 years old, 2.5–7.5%; for people aged 50–69 years old, 5–10%), i.e., 47.45% and 44.39%, respectively, and in the remaining people, it was described as very high (for people < 50 years old, ≥7.5%; for people aged 50–69 years old, ≥10%), at 8.16%. The detailed characteristics of the quantitative variables used to assess the risk of cardiovascular death are presented below (Table 2).

There are clear differences in the classification of the examined participants according to the risk classes in Pol-SCORE and SCORE2 (Table 3). A total of 33.57% of men were classified as at high or very high risk of cardiovascular death according to Pol-SCORE, while according to SCORE2, this percentage was twice as high, i.e., 66.43%. In women, these differences were even greater, i.e., 3.57% and 17.86%. The assessment of the relationship between gender and risk classes showed no significance with Pol-SCORE (Z = 1.222; *p* = 0.1414) and statistical significance in SCORE2 (Z = 6.001; *p* = 0.0000) with moderate correlation between variables (rho = 0.429; *p* = 0.0000). Approximately 85% of soldiers were classified in Pol-SCORE as at low or moderate risk of cardiovascular death, while according to SCORE2, they were classified as at low risk 59.21%. These differences are even more visible in the group of civilian employees of the army, i.e.,40.91% and 6.82%, respectively. The analyses showed statistical significance between gender and Pol-SCORE risk classes (Z = 5.015; *p* = 0.0000), with a weak correlation between variables (rho = 0.359; *p* = 0.0000) and between gender, and SCORE2 risk classes (Z = 6.316; *p* = 0.0000), with a moderate correlation between variables (rho = 0.504; *p* = 0.0000). Moreover, when analyzing the professional corps variable, a 10-fold difference in the percentage was noticed among younger officers and a 13-fold difference among senior officers classified as at low risk. In the group of non-commissioned officers according to Pol-SCORE, 30.68% had a high or very high risk of cardiovascular death, while according to SCORE2, the percentage was 62.5%. Detailed analysis indicated a statistically significant relationship between the professional corps and individual risk groups for Pol-SCORE (H = 12.170; *p* = 0.0325) and SCORE2 (H = 21.063; *p* = 0.0008). However, post hoc analysis of multiple comparisons of mean ranks for all samples showed only slight differences between the Pol-SCORE groups, i.e., non-commissioned officer, senior officer, and general or admiral, and SCORE2 groups, i.e., non-commissioned officer, senior officer, and none.

**Figure 1:**
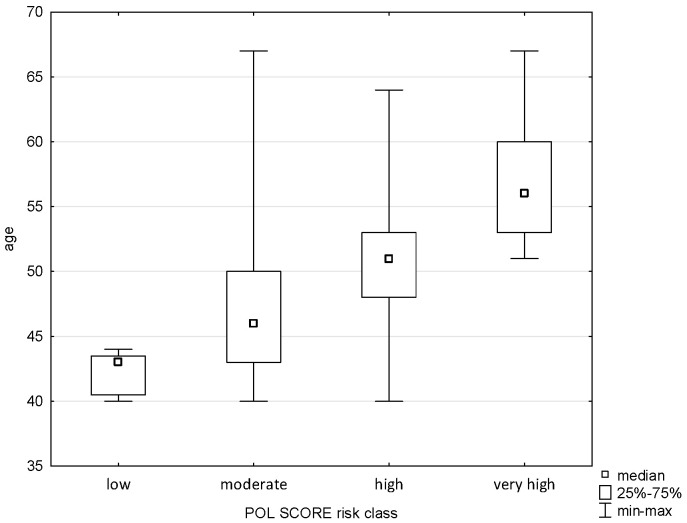
Relationship between Pol-SCORE risk class and age.

**Figure 2:**
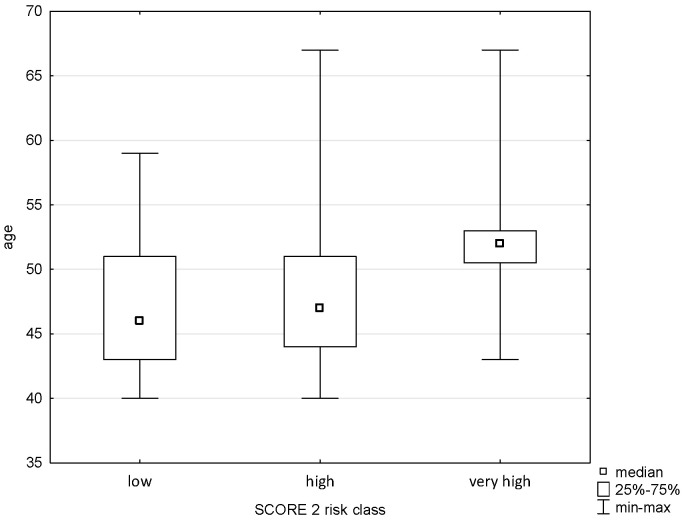
Relationship between SCORE2 risk class an age.

The applied analysis showed significant differences between the age variable and the Pol-SCORE risk classes (H = 58.719; *p* = 0.0000) and SCORE2 risk classes (H = 13.011; *p* = 0.0015). The first above-mentioned analysis indicated significant differences between all cardiovascular death risk classes, i.e., low and intermediate (*p* = 0.0005), low and high (*p* = 0.0000), low and very high (*p* = 0.00000), intermediate and high (*p* = 0.0000), and intermediate and very high (*p* = 0.0004), as shown in Figure 1. The post hoc test showed significant differences in ranks between the SCORE2 cardiovascular mortality risk groups: low and high (*p* = 0.0036) and low and very high (*p* = 0.0010), as shown in Figure 2.

**Figure 3:**
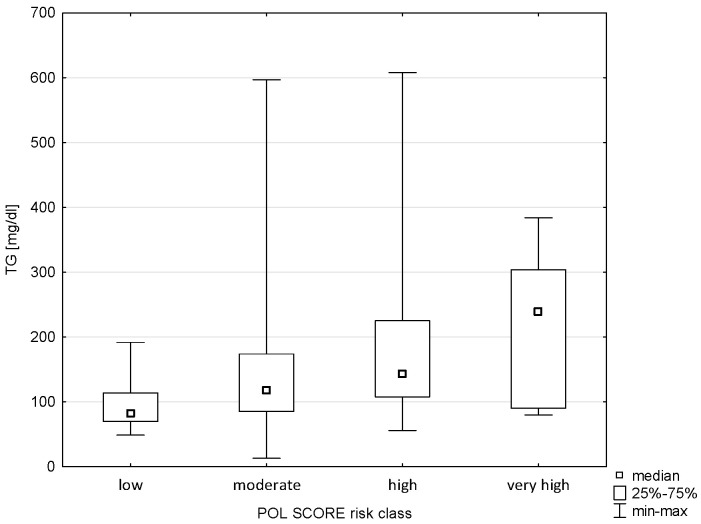
Relationship between Pol-SCORE risk class and TG.

**Figure 4:**
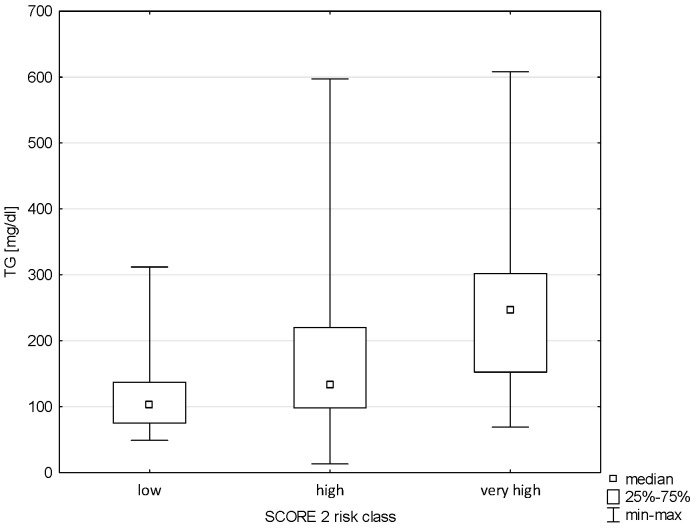
Relationship between SCORE2 risk class and TG.

A relationship was demonstrated between triglyceride levels and Pol-SCORE risk classes (H = 14.581; *p* = 0.0022) and SCORE2 risk classes (H = 29.602; *p* = 0.0000). The post hoc test showed significant rank differences between the Pol-SCORE cardiovascular death risk groups, i.e., low and high (*p* = 0.0037) and low and very high (*p* = 0.0248), as shown in Figure 3. In turn, the SCORE2 multiple comparisons analysis showed significant differences between all cardiovascular death risk groups, i.e., low and high (*p* = 0.0002), low and very high (*p* = 0.0000), and high and very high (*p* = 0.0453), as shown in Figure 4.

**Figure 5:**
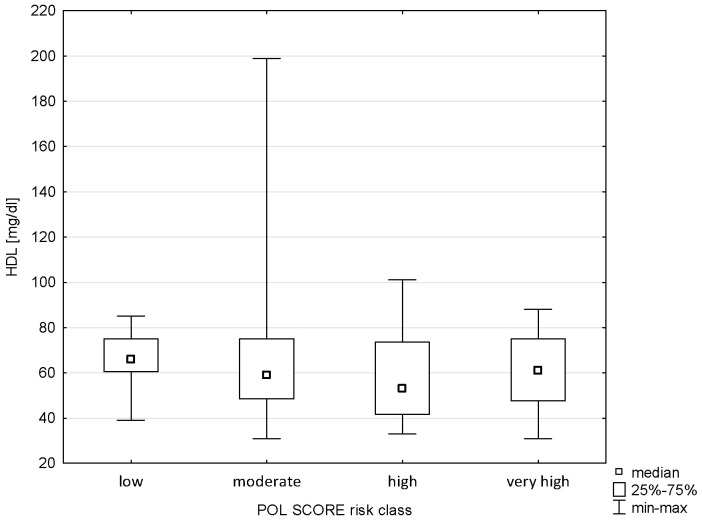
Relationship between Pol-SCORE risk class and HDL.

**Figure 6:**
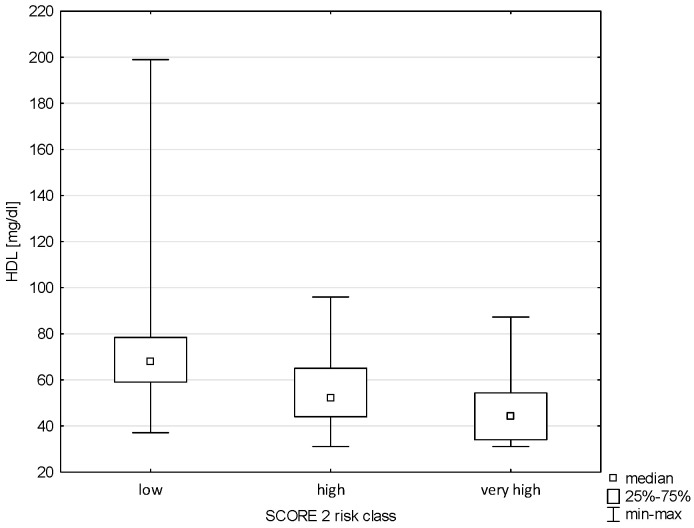
Relationship between SCORE2 risk class and HDL.

The analysis showed no correlation between Pol-SCORE risk classes and HDL cholesterol concentration (H = 5.609; *p* = 0.1322), as shown in Figure 5. In turn, significance was demonstrated between HDL and SCORE2 risk classes (H = 40.406; *p* = 0.0000). The post hoc test showed significant differences in ranks between cardiovascular death risk groups: low and high (*p* = 0.00000) and low and very high (*p* = 0.0000), as shown in Figure 6.

**Figure 7:**
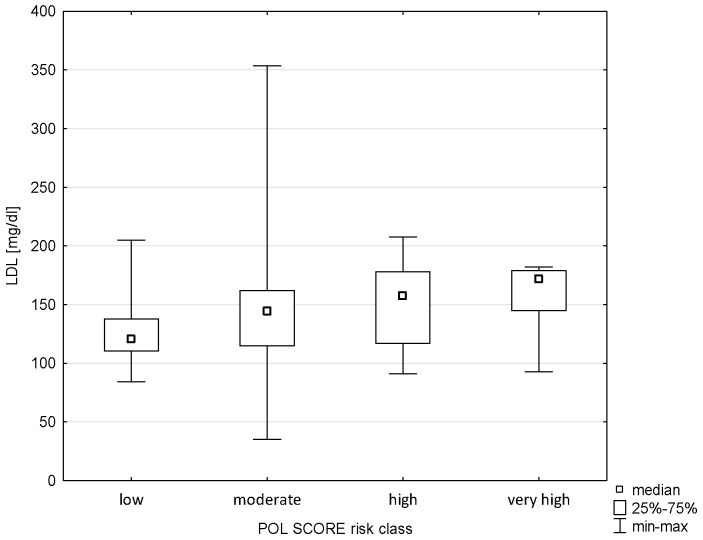
Relationship between Pol-SCORE risk class and LDL.

**Figure 8:**
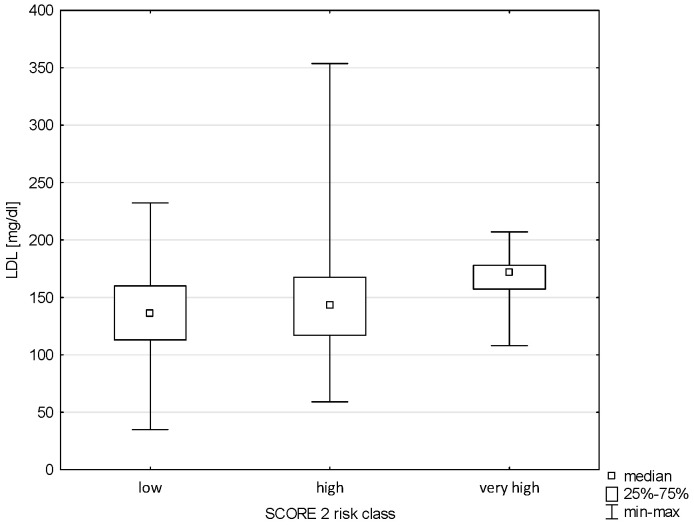
Relationship between SCORE2 risk class and LDL.

There was also no relationship between Pol-SCORE risk classes and LDL cholesterol levels (H = 7.542; *p* = 0.0565), as shown in Figure 7. In turn, significance was observed in terms of SCORE2 risk classes (H = 9.319; *p* = 0.0095). Significant differences were found between the groups of low and very high risk of cardiovascular death, i.e., *p* = 0.0073 (Figure 8).

**Figure 9:**
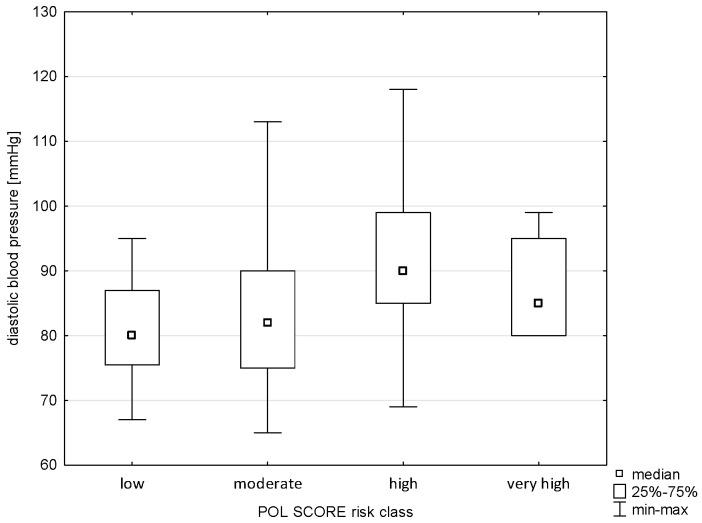
Relationship between Pol-SCORE risk class and diastolic blood pressure.

**Figure 10:**
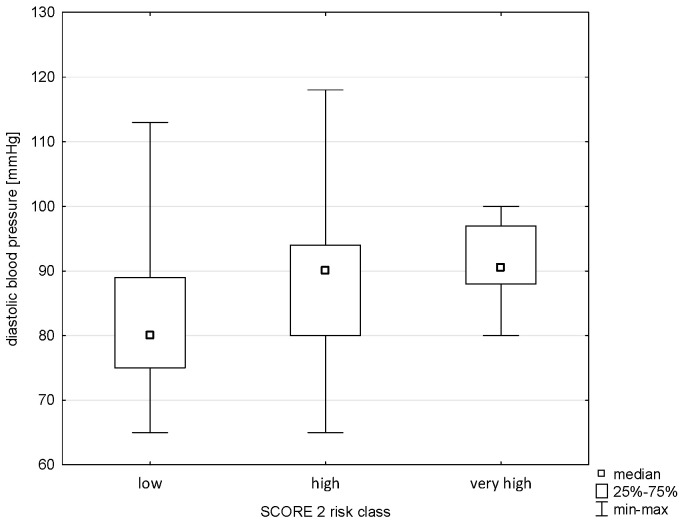
Relationship between SCORE2 risk class and diastolic blood pressure.

The applied analysis showed significant differences between the diastolic blood pressure variable and the Pol-SCORE risk classes (H = 20.441; *p* = 0.0001) and SCORE2 risk classes (H = 30.143; *p* = 0.0000). The first above-mentioned analysis indicated significant differences between the cardiovascular death risk classes, i.e., low and high (*p* = 0.0085), and medium and high (*p* = 0.0002), as shown in Figure 9. The post hoc test showed significant differences in ranks between the SCORE2 cardiovascular mortality risk groups: low and high (*p* = 0.0000) and low and very high (*p* = 0.0001), as shown in Figure 10.

For the majority of the respondents, i.e., 92.34%, including 92.67% of soldiers and 95.45% of civilian employees of the army, cardiovascular diseases and their prevention are important from the point of view of public health (Chi^2^ = 0.569; df = 2; *p* = 0.7523). Men, compared to women, more often notice the essence of the problem, i.e., 95.68% and 87.27%, respectively (Chi^2^ = 5.594; df = 2; *p* = 0.0609). A greater self-awareness of the respondents regarding the prevention of cardiovascular diseases was observed in people classified as being in a higher risk group according to Pol-SCORE and SCORE2 (Table 4); however, the analyses did not show a significant relationship (H = 2.530; *p* = 0.2821 and H = 1.259; *p* = 0.5326).

Almost half of the respondents (45.91%) had a positive family history of cardiovascular diseases, and more than one in eight had no knowledge in this area (14.28%). The occurrence of diseases in the family is definitely more common in the male population than in the female population, i.e., 61.11% and 38.89%, respectively (Chi^2^ = 11.706; df = 2; *p* = 0.0028). A total of 50.34% of soldiers and 36.36% of civilian employees of the army declared the presence of cardiovascular diseases in the family, and 34.69% of soldiers and 50.00% of civilians denied this fact (Chi^2^ = 3.520; df = 2; *p* = 0.1720). It was noted that a positive family history did not correlate significantly with the cardiovascular mortality risk classes according to Pol-SCORE (H = 3.257; *p* = 0.1962) and according to SCORE2 (H = 3.920; *p* = 0.1408). A total of 54.76% of the high Pol-SCORE class and 50.00% of the very high SCORE2 class declared no occurrence of diseases in the family, while 48.82% of the moderate Pol-SCORE class and 45.88% of the high SCORE2 class confirmed this fact (Table 5).

The frequency of selected symptoms of cardiovascular diseases was analyzed. Most respondents declared the absence of the above symptoms. Chest pain was reported by 8.38% of the respondents, including 13.46% of women and 6.47% of men (Chi^2^ = 2.406; df = 1; *p* = 0.1208) and 9.52% of soldiers and 4.55% of civilians (Chi^2^ = 1.093; df = 1; *p* = 0.2957). Dyspnea was reported by 6.28% of the population, including almost twice as often by soldiers than by civilian employees of the army, i.e., 5.44% and 9.09%, respectively (Chi^2^ = 0.765; df = 1; *p* = 0.3815), and almost eight times more often by women than by men, i.e., 17.31% and 2.16%, respectively (Chi^2^ = 17.750; df = 1; *p* = 0.0001). Dizziness was noted in 8.81% of respondents, including five times more often in women than in men, i.e., 15.38% and 3.60%, respectively (Chi^2^ = 8.289; df = 1; *p* = 0.0039). Almost no differences were observed in the occurrence of the above-mentioned symptom in soldiers and civilians (6.80% and 6.82%). The feeling of heart palpitations was reported by 13.09% of the population, including 25.00% of women and 8.63% of men (Chi^2^ = 8.911; df = 1; *p* = 0.0028). A similar percentage of soldiers (13.61%) and civilian employees (11.36%) declared the occurrence of the above-mentioned symptom (Chi^2^ = 0.149; df = 1; *p* = 0.6989). Pain in the lower limbs was declared by 11.52% of respondents, three times more often by women than men, i.e., 21.15% and 7.91%, respectively (Chi^2^ = 6.509; df = 1; *p* = 0.0107). This symptom was reported slightly more often by civilians than by soldiers, i.e., 13.64% and 10.88% (Chi^2^ = 0.251; df = 1; *p* = 0.6159). Over one-third of the population (34.55%) experienced headache, including every second (51.92%) woman and every fourth (28.06%) man (Chi^2^ = 9.531; df = 1; *p* = 0.0020). Soldiers reported the above symptom to a small extent more often than civilians, i.e., 36.05% and 29.55%, respectively (Chi^2^ = 0.634; df = 1; *p* = 0.4257). Increased blood pressure was declared by every fourth (24.08%) of the examined persons, including more often in men than in women, i.e., 26.62% and 17.31% (Chi^2^ = 1.794; df = 1; *p* = 0.1803). There were almost no differences in the occurrence of the above symptom in soldiers and civilians (24.49% and 22.73%). In most analyses divided into cardiovascular death risk classes depending on the occurrence of cardiovascular disease symptoms (Table 6), no statistical significance was noted, except for SCORE2 risk classes and the presence of headache (H = 6.549; *p* = 0.0378) and increased blood pressure (H = 6.569; *p* = 0.0374).

The frequency of selected cardiovascular risk factors was analyzed. They were present at a moderate frequency in most respondents. Overweight was reported by 33.97% of the respondents, including 22.00% of women and 38.24% of men (Chi^2^ = 4.302; df = 1; *p* = 0.0380) and 33.80% of soldiers and 34.09% of civilians (Chi^2^ = 0.001; df = 1; *p* = 0.0.9718). In turn, obesity was declared by almost every sixth (17.20%) of the examined respondents, including 22.00% of women and 15.44% of men (Chi^2^ = 1.104, df = 1; *p* = 0.2933) and 14.79% of uniformed service persons and 25.00% of civilians (Chi^2^ = 2.458; df = 1; *p* = 0.1168). Almost no differences were observed in the consumption of an inappropriate diet (43.01%), which was reported by 40.00% of women and 44.12% of men (Chi^2^ = 0.252; df = 1; *p* = 0.6150) and 42.96% of soldiers and 43.18% of civilian employees. Elevated cholesterol levels were observed in every fifth respondent (19.89%), with almost the same frequency among women (20.00%) and men (19.85%), as well as soldiers (19.01%) and civilians (22.73%). Low physical activity was declared by almost every fourth respondent (23.12%), including almost three times more often by women than men, i.e., 42.00% and 16.18% (Chi^2^ = 13.716; df = 1; *p* = 0.0002). Every fifth soldier and every third civilian also indicated the occurrence of the above-mentioned factor, i.e., 20.42% and 31.82% (Chi^2^ = 2.454; df = 1; *p* = 0.1172). Almost one-quarter of the study population (23.12%) declared smoking tobacco, with no clear difference between women and men, i.e., 18.00% and 25.00% (Chi^2^ = 1.007; df = 1; *p* = 0.3154). Civilian workers indicated smoking several times more often than soldiers, i.e., 70.45% and 8.45% (Chi^2^ = 72.658; df = 1; *p* = 0.0000). A total of 19.35% of respondents admitted to drinking alcohol, twice as often in men than in women (22.06% vs. 12.00%) and in civilians than in soldiers (31.82% vs. 15.49%). In both of the above analyses, the result was statistically insignificant, i.e., Chi^2^ = 2.369, df = 1; *p* = 0.1237 and Chi^2^ = 5.482; df = 1; *p* = 0.1397. More than half (58.06%) of the respondents indicated exposure to stress, with slight differences by occupational group, i.e., soldiers 59.15% and civilians 54.55% (Chi^2^ = 0.293; df = 1; *p* = 0.5882). In turn, in the division by gender, a significantly higher percentage of women (68.00%) than men (54.41%) indicated this risk factor (Chi^2^ = 2.772; df = 1; *p* = 0.0959). In most analyses, divided into cardiovascular death risk classes depending on the occurrence of risk factors (Table 7), statistical significance was not noted. However, a relationship was observed between the occurrence of elevated cholesterol levels and Pol-SCORE risk classes (H = 11.760; *p* = 0.0082) and SCORE2 risk classes (H = 13.108; *p* = 0.0014), with a low relationship indicated in the post hoc test only in the low and very high SCORE2 risk classes (*p* = 0.0447). The result of the relationship for low physical activity and SCORE2 risk classes also turned out to be significant (H = 6.059; *p* = 0.0483) without significant differences for multiple comparisons. Moreover, in the post hoc analysis for the variables of smoking and cardiovascular mortality risk classes, a relationship was obtained in the moderate and high risk group according to Pol-SCORE (*p* = 0.0388) as well as low and high (*p* = 0.0306) and low and very high (*p* = 0.0016) according to SCORE2.

Respondents assessed their health on a 5-point scale (5—very good; 4—good; 3—sufficient; 2—poor; 1—no opinion). More than half (59.14%) of respondents assessed their health as good, 18.62% as very good, 11.83% as satisfactory, and 0.54% as poor. There were no significant differences based on gender (Chi^2^ = 9.047; df = 4; *p* = 0.0599) or professional group (Chi^2^ = 3.404; df = 4; *p* = 0.4926). Men were much more likely to describe their health as very good compared to women, i.e., 20.59% vs. 14.00%. A much larger percentage of women (20.00%) than men (5.88%) had no opinion on their health. In turn, when divided into professional groups, soldiers more often than civilian employees assessed their health as very good, i.e., 27.27% vs. 16.20%. A slightly higher percentage of civilians (12.68%) than soldiers (9.09%) had a bad opinion about their health. In the conducted analysis, no correlation was observed between the affiliation to the cardiovascular death risk classes according to Pol-SCORE (H = 1.577; *p* = 0.8128) and SCORE2 (H = 2.230; *p* = 0.6934) and the self-assessment of the health status of the respondents (Table 8).

The respondents showed insufficient care for their own health, and only one in four (27.81%) of them performed preventive examinations to detect cardiovascular diseases once a year, and one in eight (12.30%) performed them once every 5 years. No significant differences were observed in the division by gender (Chi^2^ = 5.534; df = 5; *p* = 0.3541), with a three times higher percentage of men than women undergoing examinations once a year (33.82% vs. 11.76%) and a slightly higher percentage of women than men undergoing the above examinations more often than once a year (15.69% vs. 11.03%). Most often, the tests are performed as part of periodic examinations by an occupational medicine doctor (41.18%). Also, in the division by professional group, no statistically significant differences were observed (Chi^2^ = 5.534; df = 5; *p* = 0.3541). Almost the same percentage of soldiers and civilian employees of the army performed preventive examinations once a year or did not perform them at all, i.e., 28.67% and 25.00% and 4.90% vs. 4.55%, respectively. A slightly higher percentage of civilians than uniformed employees were tested only during periodic examinations, i.e., 45.45% vs. 39.86%. In the analysis (Table 9), no statistical correlation was observed between the frequency of preventive examinations for cardiovascular diseases and the risk classes of death from cardiovascular causes according to Pol-SCORE (H = 8.467; *p* = 0.1323) and SCORE2 (H = 7.974; *p* = 0.1577).

## 4. Discussion

This study aimed to compare cardiovascular risk classification outcomes in a military population using two tools: the Polish adaptation of SCORE and the newer SCORE2 model.

The comparison between the Pol-SCORE and SCORE2 tools revealed significant differences in cardiovascular risk stratification among military personnel, which may have important implications for clinical decision-making in this specific population.

However, the MIL-SCORE program revealed concerning indicators: 60.00% of soldiers had elevated LDL cholesterol levels, 36.00% had hypertriglyceridemia, and 44.70% had hypertension. The percentage of obese individuals increased with age, reaching 27.00% among soldiers over the age of 50, while 58.00% were classified as overweight. These findings challenge the stereotype that soldiers are always in optimal physical condition and highlight the need for routine screening focused on metabolic parameters in the armed forces [1]. Similar results were obtained in the present study, where the mean systolic blood pressure was 138.98 (±15.96) mmHg and the mean total cholesterol level was 231.01 (± 44.79) mg/dL.

In the present study, significant differences were observed in the proportions of individuals classified as being at high and very high risk depending on the model used: 33.57% of men according to Pol-SCORE and 66.43% according to SCORE2. Clear differences in risk levels were also visible between soldiers and civilian military employees, especially in younger age groups and those with higher military ranks. Similar discrepancies were observed by Boskovic et al., who examined a group of 1317 patients (non-military) aged 40 to 70 years without diagnosed cardiovascular disease. Based on the SCORE model, 166 patients (12.60%) were classified as low risk, 658 (49.90%) as moderate risk, 276 (20.9%) as high risk, and 217 (16.60%) as very high risk. In contrast, based on the SCORE2 model, 30 patients (2.80%) were classified as low-to-moderate risk, 273 (18.00%) as high risk, and 1014 (79.20%) as very high risk. Under SCORE2, significantly fewer patients were classified in the low-to-moderate risk group compared to SCORE (30; 2.80% vs. 824; 62.60%; *p* < 0.001), while significantly more were placed in the very high risk group (1014; 79.20% vs. 217; 16.60%; *p* < 0.001). No significant differences were observed in the number of patients classified as high risk [17].

These discrepancies are in line with findings by Csenteri et al., who reported that the application of SCORE2 instead of the previous SCORE algorithm resulted in 43.91% of the total study population being reclassified into a higher risk category, indicating a significant increase in the number of patients identified as being at high or very high cardiovascular risk [16]. Building on these observations, further analysis revealed that demographic variables such as age and sex significantly influenced risk classification outcomes. When the results were stratified by age group, marked differences were observed between SCORE and SCORE2. According to the SCORE model, 97.70% of men aged 40–50 years were classified as low-to-moderate risk, whereas this proportion dropped to 32.40% under SCORE2. Among women in the same age group, SCORE classified 100.00% as low-to-moderate risk, while SCORE2 assigned 75.60% to this category. In the 50–65 age group, SCORE identified 36.80% of men as high risk and 14.80% as very high risk; for women, 5.40% were classified as high risk and 0.50% as very high risk. In contrast, using SCORE2, 50.00% of men in this age group were categorized as high risk and 25.80% as very high risk. Among women, 38.80% were classified as high risk and 11.90% as very high risk [16]. In our analyses, a total of 33.57% of men were classified as at high or very high risk of cardiovascular death according to Pol-SCORE, whereas this figure doubled under SCORE2, reaching 66.43%. Among women, the difference was even more pronounced—3.57% versus 17.86%. While the association between sex and risk categories was not statistically significant in Pol-SCORE, it reached statistical significance when evaluated using SCORE2. A Polish study comparing the SCORE2 model with the regionally calibrated Pol-SCORE model in 159 military patients showed that SCORE2 estimated the 10-year risk of cardiovascular events as twice as high compared to the mortality risk assessed by Pol-SCORE. While Pol-SCORE assigned 65.41% of participants to the moderate risk category, SCORE2 placed 49.06% in the low-to-moderate risk range, suggesting greater precision in event prediction [18]. These findings reflect broader trends observed in European and international studies validating the predictive strength of SCORE2. The estimated 10-year risk calculated using this model falls between the values for high and low risk groups under SCORE and increases significantly with age. Very few individuals under the age of 50 have a 10-year risk above 5.00% (the European threshold for high risk). More than half of men aged 60 have an estimated risk above this threshold, whereas only 7.00% of women at the same age exceed it. Even when the risk threshold is lowered to 1.00% for younger age groups, very few women under 50 exceed this level [19]. In Russia, where cardiovascular risk is considered very high, according to the SCORE2 model, no man would be classified as having a “low to moderate” 10-year risk [20].

In addition to the tool comparison, our results should be interpreted within the broader context of the health status and risk profile of military personnel. Our data confirmed similar discrepancies in cardiovascular risk stratification depending on military rank. For example, among private soldiers, SCORE2 classified three times more individuals as high risk compared to Pol-SCORE (75.00% vs. 25.00%). In the group of junior officers, SCORE2 indicated a more polarized distribution, with an increase in both low- and high-risk classifications. Among senior officers, the proportion of individuals in the low-risk group increased 13-fold under SCORE2 compared to Pol-SCORE (38.24% vs. 2.94%). These findings suggest that the application of the SCORE2 model leads to reclassification of risk categories, particularly increasing the proportion of individuals identified as high or very high risk, especially among private soldiers and senior officers, highlighting the potential of SCORE2 to better reflect contemporary cardiovascular risk in the military population.

A retrospective cohort study on trends in the incidence of CVD and six risk factors (per 10,000 individuals) among active-duty soldiers, based on data from the Defense Medical Epidemiology Database (DMED) from 2016–2021, showed a significant increase in poor dietary habits—by nearly 55.00%—and a decrease in overweight/obesity by 21%, hypertension by 30.00%, hypercholesterolemia by 24%, and smoking by almost 37.00% [21]. Elevated total cholesterol (TC) in the blood has long been one of the primary criteria for assessing cardiovascular health in both children and adults and is a major risk factor for the development of atherosclerosis and cardiovascular diseases [7]. Measuring this parameter should not present any difficulty, and in cases with a family history of cardiovascular disease, this test should be performed at least once a year. The study by Gielerak et al. [1] showed that Polish soldiers exhibit many cardiovascular (CV) risk factors, similar to those observed in the general population. The results of our own study also support these observations. Our analysis revealed elevated blood pressure in 24.08% of respondents, more frequently among men than women (26.62% vs. 17.31%). Significant differences were noted in diastolic blood pressure values across cardiovascular risk classes according to both the Pol-SCORE and SCORE2 models. Very high risk was observed in 28.6% of participants under Pol-SCORE and 18.75% under SCORE2. There were virtually no differences in the occurrence of this symptom between soldiers and civilian employees (24.49% vs. 22.73%). In turn, a Czech study found that male soldiers were significantly more burdened with CV risk factors compared to the civilian population [22]. Zimmerman, in turn, pointed out that CV risk factors contribute to high mortality among law enforcement officers [23]. Among police officers in Quebec, 9.00% of deaths were attributed to cardiovascular diseases, despite their young age [24].

Modifiable lifestyle and psychosocial factors also played a critical role. The nature of military service or uniformed work is inherently associated with prolonged stress and functioning under extreme conditions. These factors may exacerbate adverse health effects and increase the risk of CVD. The latest studies indicate that stress was the most frequently reported cardiovascular risk factor in this occupational group. A high level of stress was recorded in over 50.00% of individuals in the high and very high CVD risk groups according to SCORE—highlighting the need to implement psychological support programs and stress management strategies in the workplace or service environment [25]. An unhealthy diet, lack of physical activity, and chronic stress are also leading causes of hypertension, which affects over 13.00% of Polish soldiers, while another 16.00% fall into a high risk group [1]. Soldiers, as an occupational group, often operate under high-stress conditions. Moreover, some of them enter military service already having experienced difficult life circumstances, such as childhood trauma, which can be exacerbated by service-related stressors. A report by the American Heart Association highlighted an interesting relationship between stress and smoking: 28.10% of smokers reported experiencing severe psychological stress compared to 10.90% of non-smokers [7]. These data, however, refer to civilians. Our study indicates that stress affects nearly 60.00% of soldiers and 54.55% of civilian military employees. This rate is linked to the specific nature of military service, which is associated with high exposure to specific occupational stressors (e.g., harsh living conditions and deployment in combat operations), all of which contribute to an increased risk of cardiovascular disease. Psychological stress emerged as one of the most critical non-biological risk factors in our population. In a study involving 8727 soldiers who had experienced traumatic injuries, it was shown that they were more likely to develop hypertension, diabetes, and coronary artery disease compared to soldiers without such injuries [21]. In our study, 23.12% of participants reported tobacco use, with a higher prevalence among men (25.00%) than women (18.00%). Notably, civilian employees were several times more likely to smoke than soldiers, with prevalence rates of 70.45% and 8.45%, respectively. Other studies among Polish soldiers indicate that more than 72.00% of soldiers aged 20–30 years are regular smokers, although this percentage decreases with age—among soldiers aged 50+, approximately 41.00% still smoke [18], which remains a high rate. Among military personnel in Lithuania, the current smoking rate is 45.90% among men and 17.90% among women. The stress smoking prevalence was observed in men aged 35–44 years (64.5%) [26]. According to the 2021 National Health Interview Survey, 18.7% of adults in the United States reported current use of any commercial tobacco product. While the prevalence of traditional cigarette smoking has declined, the use of e-cigarettes has increased (from 3.70% to 4.50% among adults). Smoking not only decreases physical fitness but also increases the risk of heart attack and stroke. The problem is so significant that in 2011, the Polish Ministry of National Defence (MON) launched a prevention campaign. In cooperation with the Social Education Foundation, the ministry developed a plan to combat smoking in the military [27]. Currently (April 2025), work is underway on a draft regulation concerning the specific conditions for the use of tobacco products on the premises of institutions subordinate to or supervised by the Ministry of National Defence and in means of transport used by these entities. The draft regulation addresses the need to protect non-smokers and maintain workplaces, educational environments, and service areas as smoke-free zones, free from emissions of tobacco products, heated tobacco devices, or vapor from electronic cigarettes [28].

Another factor influencing the occurrence of cardiovascular diseases (CVD) is low physical activity. A total of 23.12% of participants reported insufficient physical activity. Depending on the risk assessment model, this proportion was 35.70% for Pol-SCORE and 25.60% for SCORE2. For comparison, among younger soldiers aged 20–30 years, only slightly more than 9.00% reported low activity, indicating a marked increase with age or over time. This rate increased with age—nearly 30.00% of soldiers aged 50+ admitted to low levels of physical activity [1]. Data from the National Health Interview Survey show that, depending on the state, between 17.70% and as many as 49.40% of adults aged ≥18 years reported no leisure-time physical activity in the past month [7]. Considering that the latest recommendations indicate only 75 min of vigorous aerobic exercise per week—as an alternative to the previous recommendation of at least 2.5 h per week of moderate aerobic activity [10]—such low levels of physical activity may significantly contribute to the increasing risk of cardiovascular diseases, including hypertension, atherosclerosis, coronary artery disease, and premature cardiovascular mortality.

Research confirms that lower levels of physical activity and a higher prevalence of tobacco use among soldiers may, among other factors, be associated with sleep deprivation, which is likely the most neglected pillar of lifestyle medicine among current members of the armed forces. This neglect is probably due to the historical underappreciation of sleep within military tradition [29]. When interpreting the results, particular attention should be paid to specific occupational groups, such as uniformed services and soldiers. The military is largely composed of young individuals. Nearly 90.00% of candidates enlist in the armed forces between the ages of 17 and 24 [29]. For this reason, soldiers are often perceived as physically fit and generally healthy at the beginning of their professional careers [30]. A 2024 review encompassing 12 systematic reviews, including 53 meta-analyses, over 500 cohort studies, and 12 analyses, shows that each 5 kg/m^2^ increase in body mass index (BMI) is associated with an increased risk of stroke, coronary artery disease, atrial fibrillation, heart failure, and hypertension [7]. Data from the National Health Interview Survey indicate that 71.20% of adults aged ≥20 years were overweight or obese, including 41.90% with obesity [7]. Projections from the World Obesity Federation are equally concerning: by 2035, over half of the world’s population (51.00%), or more than 4 billion people, are expected to be overweight or obese. Among them, one in four individuals—nearly 2 billion—will be living with obesity [31]. Nearly 7 in 10 active-duty soldiers in the U.S. military are overweight or obese based on BMI [32]. Our study supports these findings, revealing a high prevalence of factors such as overweight (33.97%) and obesity (17.20%). According to Martin, although individuals who are overweight tend to have a similar life expectancy to those with a normal BMI, the increased risk of early development of cardiovascular diseases leads to a greater number of years lived with illness [7].

While most participants reported being aware of preventive measures, a notable disconnect was observed between perceived and actual cardiovascular risk. This gap was particularly evident in self-assessed health status and preventive care behaviors. Although no significant associations were found between self-assessment of health status and cardiovascular risk classes according to either Pol-SCORE or SCORE2, the general lack of preventive care is concerning. Only 27.81% of respondents underwent annual preventive screenings, highlighting the need for health education. A large-scale study involving more than 21,000 new U.S. Army recruits showed that subjective health assessments, combined with detailed questionnaire data, can serve as an effective tool for identifying individuals at elevated health risk (e.g., suicide attempts, psychiatric hospitalizations, injuries, violence) [33]. In our study, more than half of the respondents assessed their health as “good” (59.14%), despite the fact that 88.27% had moderate or high risk according to Pol-SCORE and 44.39% had high risk according to SCORE2. A cross-sectional study among 3874 New Zealand military veterans also confirmed that soldiers’ self-rated health is strongly associated with experienced health problems, both physical and mental, as well as with sociodemographic factors [34].

Although 92.34% of respondents declared awareness of preventive actions, this did not translate into sufficient screening behaviors. More than one in eight respondents (14.28%) lacked knowledge regarding the presence of cardiovascular disease in their family. Moreover, the prevalence of selected cardiovascular risk factors among respondents was moderate: 13.09% reported heart palpitations, 8.38% reported chest pain, and 6.28% experienced shortness of breath. These findings further confirm that cardiovascular diseases often develop asymptomatically and remain undetected for a long time. Therefore, it seems essential to place greater emphasis on preventive screening and public education in this area.

Overall, the SCORE2 model appears to more accurately identify individuals at increased cardiovascular risk within the military population. Given the unique occupational profile of military personnel, accurate cardiovascular risk stratification must combine robust tools like SCORE2 with a nuanced understanding of modifiable factors such as stress, physical activity, and smoking. Only such an approach can lead to meaningful prevention and improved long-term outcomes in this population.

The strengths of the study include the following:

1. The use of two validated cardiovascular risk assessment models (Pol-SCORE and SCORE2) in the same cohort, which allows for a direct comparison of their performance;
2. The focus on a unique and under-researched population (military personnel and civilian employees of the armed forces), where cardiovascular health is of particular importance;
3. The standardized assessment of major cardiovascular risk factors, including blood pressure, lipid profile, BMI, and self-reported lifestyle and stress indicators;
4. The relevance of the findings for both clinical practice and occupational medicine, providing evidence that may inform future preventive strategies in the armed forces.

The main limitation of our study is the lack of lipoprotein(a) [Lp(a)] measurement, which is now recommended at least once per lifetime according to the latest European guidelines. At the time of data collection, Lp(a) testing was not routinely available in the military medical setting, which prevented its inclusion. Future research in this population should incorporate Lp(a) assessment to enable more comprehensive cardiovascular risk stratification.

Another limitation is the cross-sectional design, which does not allow for causal inference or the assessment of long-term outcomes. Longitudinal studies are needed to verify whether the observed differences in risk classification between Pol-SCORE and SCORE2 translate into actual differences in cardiovascular events.

A further limitation is the relatively small sample size (*n* = 196), which may restrict the generalizability of the results, particularly when interpreting subgroup analyses by gender or military rank. Additionally, the study population was racially homogeneous, which may limit the applicability of our findings to other races and ethnicities.

Finally, the study population consisted exclusively of middle-aged military personnel and civilian employees (40–67 years). Therefore, the findings cannot be directly extrapolated to younger soldiers or to other populations outside the military context.

Future directions: we highlight the need for longitudinal follow-up studies, larger cohorts including younger soldiers, and the incorporation of additional biomarkers such as Lp(a) and environmental stressors to better understand cardiovascular risk in this population.

## 5. Conclusions

The study indicates that the Pol-SCORE and SCORE2 tools yield different results in cardiovascular risk classification among military personnel. Significant differences were observed, particularly in risk distribution and stress levels. This highlights the need to select appropriate risk assessment tools tailored to specific populations, such as soldiers and civilian employees of the armed forces.

## Figures and Tables

**Table 1:** Differences between ACC/AHA and ESC/ESH guidelines on arterial hypertension. ACC = American College of Cardiology; ACE = angiotensin-converting enzyme; AHA = American Heart Association; BP = blood pressure; ESC = European Society of Cardiology; ESH = European Society of Hypertension [10,11].

Guideline Differences	2017 ACC/AHA	2024 ESC/ESH
hypertension definition	≥130/80	≥130/80
normal BP ranges (mmHg)	Normal: <120/80 Elevated: 120–129/<80	Optimal: <120/80 Normal: 120–129/80–84 High–Normal: 130–139/85–89
hypertensive BP ranges (mmHg)	Hypertension Stage 1: 130–139/80–89 Hypertension Stage 2: ≥140/90	Hypertension Grade 1: 14–159/90–99 Hypertension Grade 2: 160–179/100–109 Hypertension Grade 3: ≥180/110
BP targets for treatment		
18–64 years (mmHg)	<130/80	<130/80
65–79 years (mmHg)	<130/80	<140/80
≥80 years (mmHg)	<130/80	140–150/<80

**Table 2:** Characteristics of the study participants in general and by cardiovascular mortality risk classes according to Pol-SCORE and SCORE2.

Variables			$\bar{x}$	SD	Me	Min	Max
TOTAL		systolic blood pressure	138.98	15.96	136.00	104.00	190.00
		total cholesterol	231.01	44.79	225.00	121.00	459.60
		non-HDL cholesterol	169.10	44.60	168.00	50.00	380.20
		age	47.83	5.32	47.00	40.00	67.00
Pol-SCORE	LOW	systolic blood pressure	131.69	9.02	132.5	115.00	145.00
		total cholesterol	212.6	31.77	203.00	172.20	304.00
		non-HDL cholesterol	145.49	33.28	139.65	94.10	235.00
		age	42.19	1.60	43.00	40.00	44.00
	MODERATE	systolic blood pressure	135.69	13.99	135.00	104.00	170.00
		total cholesterol	228.69	46.45	228.00	121.00	459.60
		non-HDL cholesterol	166.00	45.22	166.00	50.00	380.00
		age	46.98	4.71	46.00	40.00	67.00
	HIGH	systolic blood pressure	149.04	17.59	150.00	114.00	190.00
		total cholesterol	240.64	40.78	234.85	167.00	321.00
		non-HDL cholesterol	183.07	40.42	182.65	107.7	253.00
		age	51.07	4.38	51.00	40.00	64.00
	VERY HIGH	systolic blood pressure	157.00	15.69	150.00	140.00	189.00
		total cholesterol	258.60	44.85	268.00	185.60	323.60
		non-HDL cholesterol	197.10	49.54	209.00	110.70	248.60
		age	57.00	5.50	56.00	51.00	67.00
SCORE2	LOW	systolic blood pressure	131.94	11.60	133.00	104.00	170.00
		total cholesterol	225.09	43.97	220.00	121.00	340.00
		non-HDL cholesterol	154.80	41.68	153.00	50.00	273.00
		age	47.09	4.82	46.00	40.00	59.00
	HIGH	systolic blood pressure	144.29	16.67	142.00	112.00	190.00
		total cholesterol	232.17	45.66	222.20	130.00	459.60
		non-HDL cholesterol	176.56	42.89	170.10	75.00	380.20
		age	47.74	5.43	47.00	40.00	67.00
	VERY HIGH	systolic blood pressure	151.06	16.24	150.00	119.00	189.00
		total cholesterol	259.06	34.67	265.00	168.00	323.60
		non-HDL cholesterol	211.65	33.78	216.20	135.00	253.00
		age	52.50	5.42	52.00	43.00	67.00

**Table 3:** Characteristics of the participants divided into Pol-SCORE risk classes and SCORE2 risk classes.

Variables		Pol-SCORE				SCORE2		
		Low	Moderate	High	Very High	Low	High	Very High
gender [%]	male	-	66.43	28.57	5.00	33.57	55.71	10.72
	female	28.57	67.86	3.57	-	82.14	16.07	1.79
study group [%]	soldier	10.53	74.34	14.47	0.66	59.21	38.82	1.97
	civilian worker	40.91	-	45.45	13.64	6.82	63.64	29.55
military services corps [%]	private soldier	12.50	62.50	25.00	-	25.00	75.00	-
	non-commissioned oficer	5.68	63.64	28.41	2.27	37.50	53.41	9.09
	junior oficer	5.00	70.00	20.00	5.00	50.00	45.00	5.00
	senior oficer	2.94	67.65	20.59	8.82	38.24	44.12	17.64
	general, admiral	-	66.67	33.33	-	66.67	33.33	-
	none	18.60	72.09	6.98	2.33	76.74	20.93	2.33

**Table 4:** Self-awareness of respondents regarding the prevention of cardiovascular diseases divided into risk classes in Pol-SCORE and SCORE2.

Variables		Pol-SCORE				SCORE2		
		Low	Moderate	High	Very High	Low	High	Very High
self-awareness in preventing cardiovascular diseases	yes	86.67	92.31	97.62	100.00	92.39	93.02	100.00
	no	13.33	7.69	2.38	-	7.61	6.98	-

**Table 5:** Occurrence of cardiovascular diseases in the family divided into Pol-SCORE and SCORE2 risk classes.

Variables		Pol-SCORE				SCORE2		
		Low	Moderate	High	Very High	Low	High	Very High
occurrence of cardiovascular diseases in the family	yes	66.67	48.82	35.72	42.86	51.11	45.88	31.25
	no	20.00	34.65	54.76	42.86	31.11	43.53	50.00
	no opinion	13.33	16.53	9.52	14.28	17.78	10.59	18.75

**Table 6:** Occurrence of cardiovascular disease symptoms in the study participants divided into Pol-SCORE and SCORE2 risk classes.

Variables		Pol-SCORE				SCORE2		
		Low	Moderate	High	Very High	Low	High	Very High
chest pain	yes	6.67	10.24	4.76	-	11.11	7.06	-
	no	93.33	89.76	95.24	100.00	88.89	92.94	100.00
Statistics		H = 1.973; *p* = 0.5778				H = 2.518; *p* = 0.2839		
dyspnoea	Yes	13.33	6.30	2.38	14.29	8.89	2.35	12.50
	No	86.67	93.70	97.62	85.71	91.11	97.65	87.50
Statistics		H = 3.097; *p* = 0.3768				H = 4.295; *p* = 0.1168		
dizziness	Yes	-	8.66	4.76	-	7.78	5.88	6.25
	No	100.00	91.34	95.24	100.00	92.22	94.12	93.75
Statistics		H = 2.559; *p* = 0.4647				H = 0.254; *p* = 0.8804		
heart palpitations	Yes	13.33	14.17	7.14	28.57	15.56	10.59	12.50
	No	86.67	85.83	92.86	71.43	84.44	89.41	87.50
Statistics		H = 2.897; *p* = 0.4078				H = 0.948; *p* = 0.6224		
pain in the lower limbs	Yes	6.67	13.39	7.14	14.29	15.56	5.88	18.75
	No	93.33	86.61	92.86	85.71	84.44	94.12	81.25
Statistics		H = 1.614; *p* = 0.6562				H = 4.883; *p* = 0.0870		
headache	Yes	60.00	35.43	26.19	14.29	42.22	24.71	43.75
	No	40.00	64.57	73.81	85.71	57.78	75.29	56.25
Statistics		H = 6.872; *p* = 0.0761				H = 6.549; *p* = 0.0378		
increased blood pressure	Yes	-	24.41	30.95	28.57	16.67	32.94	18.75
	No	100.00	75.59	69.05	71.43	83.33	67.06	81.25
Statistics		H = 5.895; *p* = 0.1168				H = 6.569; *p* = 0.0374		

**Table 7:** Prevalence of cardiovascular risk factors divided into Pol-SCORE and SCORE2 classes.

Variables		Pol-SCORE				SCORE2		
		Low	Moderate	High	Very High	Low	High	Very High
overweight	Yes	14.29	34.15	40.48	28.57	27.91	36.09	50.00
	No	85.71	65.85	59.52	71.43	72.09	63.10	50.00
statistics		H = 3.289; *p* = 0.3491				H = 3.549; *p* = 0.1695		
obesity	Yes	14.29	14.63	26.19	14.29	13.95	16.67	37.50
	No	85.71	85.37	73.81	85.71	86.05	83.33	62.50
statistics		H = 3.060; *p* = 0.3824				H = 5.253; *p* = 0.0723		
incorrect diet	Yes	42.86	41.46	47.62	42.86	40.70	41.67	62.50
	No	57.14	58.54	52.38	57.14	59.30	58.33	37.50
statistics		H = 0.481; *p* = 0.9229				H = 2.714; *p* = 0.2574		
increased cholesterol levels	Yes	7.14	15.45	38.10	14.29	11.63	22.62	50.00
	No	92.86	84.55	61.90	85.71	88.37	77.38	50.00
statistics		H = 11.760; *p* = 0.0082				H = 13.108; *p* = 0.0014		
low physical activity	Yes	35.71	20.33	28.57	14.29	25.58	16.67	43.75
	No	64.29	79.67	71.43	85.71	74.42	83.33	56.25
statistics		H = 2.784; *p* = 0.4261				H = 6.059; *p* = 0.0483		
smoking	Yes	7.14	17.07	45.24	28.57	8.14	30.95	62.50
	No	92.86	82.93	54.76	71.43	91.86	69.05	37.50
statistics		H = 16.131; *p* = 0.0011				H = 27.569; *p* = 0.0000		
drinking alcohol	Yes	7.14	17.07	30.95	14.29	15.12	20.24	37.50
	No	92.86	82.93	69.05	85.71	84.88	79.76	62.50
statistics		H = 5.452; *p* = 0.1415				H = 4.383; *p* = 0.1117		
stress	Yes	71.43	59.35	54.76	28.57	60.47	55.95	56.25
	no	28.57	40.65	45.24	71.43	39.53	44.05	43.75
statistics		H = 3.778; *p* = 0.2864				H = 0.377; *p* = 0.8282		

**Table 8:** Self-assessment of the health status of the respondents divided into risk classes in Pol-SCORE and SCORE2.

Variables		Pol-SCORE				SCORE2		
		Low	Moderate	High	Very High	Low	High	Very High
self-assessment of health	very good	26.67	18.03	16.67	28.57	18.60	19.05	18.75
	good	53.33	59.84	61.90	42.86	58.14	60.72	56.25
	sufficient	13.33	10.66	11.90	28.57	10.47	11.90	18.75
	poor	-	0.81	-	-	1.16	-	-
	no opinion	6.67	10.66	9.53	-	11.63	8.33	6.25

**Table 9:** Frequency of performing preventive examinations for cardiovascular diseases divided into risk classes in Pol-SCORE and SCORE2.

Variables		Pol-SCORE				SCORE2		
		Low	Moderate	High	Very High	Low	High	Very High
frequency of preventive tests for cardiovascular diseases	more than once a year	-	8.13	2.38	-	8.05	4.76	-
	once a year	6.67	23.57	42.86	57.14	18.39	35.71	37.50
	once every 5 years	13.33	12.20	9.52	28.57	13.79	10.71	12.50
	when I have symptoms	20.00	8.13	4.76	-	8.05	8.33	6.25
	only periodic tests at work	46.67	42.28	40.48	14.29	45.98	35.71	43.75
	I do not get tested	13.33	5.69	-	-	5.74	4.76	-

## Data Availability

The original contributions presented in this study are included in the article/Supplementary Materials. Further inquiries can be directed to the corresponding author.

## Supplementary Materials

The following supporting information can be downloaded at: https://www.mdpi.com/article/10.3390/pathophysiology32030045/s1, Supplementary File S1: anonymous survey.

## References

[1] Gielerak G., Krzesiński P., Piotrowicz K., Murawski P., Skrobowski A., Stańczyk A., Galas A., Uziębło-Życzkowska B., Kaźmierczak-Dziuk A., Maksimczuk J. (2020). The Prevalence of Cardiovascular Risk Factors among Polish Soldiers: The Results from the MIL-SCORE Program. Cardiol. Res. Pract..

[2] Di Cesare M., Perel P., Taylor S., Kabudula C., Bixby H., Gaziano T.A., McGhie D.V., Mwangi J., Pervan B., Narula J. (2024). The Heart of the World. Glob. Heart.

[3] Yusuf S., Hawken S., Ôunpuu S., Dans T., Avezum A., Lanas F., McQueen M., Budaj A., Pais P., Varigos J. (2004). Effect of potentially modifiable risk factors associated with myocardial infarction in 52 countries (the INTERHEART study): Case-control study. Lancet.

[4] Perone F., Bernardi M., Spadafora L., Betti M., Cacciatore S., Saia F., Fogacci F., Jaiswal V., Asher E., Paneni F. (2025). Non-Traditional Cardiovascular Risk Factors: Tailored Assessment and Clinical Implications. J. Cardiovasc. Dev. Dis..

[5] Polska Akademia Nauk (2021). Antynomie Systemu Ochrony Zdrowia.

[6] Agencja Oceny Technologii Medycznych i Taryfikacji (2023). Rekomendacja nr 1/2023 Prezesa AOTMiT z 29 Grudnia 2023 r. w Sprawie Zalecanych Technologii Medycznych, Działań Przeprowadzanych w Ramach Programów Polityki Zdrowotnej Oraz Warunków Realizacji Tych Programów, Dotyczących Profilaktyki Uzależnień od Tytoniu (Nikotyny). https://www.aotm.gov.pl.

[7] Martin S.S., Aday A.W., Almarzooq Z.I., Anderson C.A., Arora P., Avery C.L., Baker-Smith C.M., Gibbs B.B., Beaton A.Z., Boehme A.K. (2024). 2024 Heart Disease and Stroke Statistics: A Report of US and Global Data From the American Heart Association. Circulation.

[8] Lach J., Bzdęga J., Kubiak L., Ostalska J., Żbikowski J., Nieporęcki R. (2014). Wojskowe zakłady lecznicze armii polskiej. Część I. Chorzy leczeni w latach 1922–1931 na podstawie sprawozdania gen. Stanisława Roupperta do Marszałka Józefa Piłsudskiego. Hygeia Public Health.

[9] Trzeciak B.G., Kowalczyk W., Grymek S., Gutknecht P., Siebert J. (2023). Cardiovascular risk factors among Polish employees of uniformed services. Int. J. Occup. Med. Environ. Health.

[10] Vemu P.L., Yang E., Ebinger J. (2024). 2023 ESH Hypertension Guideline Update: Bringing Us Closer Together Across the Pond.

[11] Vrints C., Andreotti F., Koskinas K.C., Rossello X., Adamo M., Ainslie J., Banning A.P., Budaj A., Buechel R.R., Chiariello G.A. (2024). 2024 ESC Guidelines for the management of chronic coronary syndromes. Eur. Heart J..

[12] De Backer G. (2003). European guidelines on cardiovascular disease prevention in clinical practice Third Joint Task Force of European and other Societies on Cardiovascular Disease Prevention in Clinical Practice (constituted by representatives of eight societies and by invited experts). Eur. Heart J..

[13] Conroy R. (2003). Estimation of ten-year risk of fatal cardiovascular disease in Europe: The SCORE project. Eur. Heart J..

[14] SCORE2 Working Group and ESC Cardiovascular Risk Collaboration (2021). SCORE2 risk prediction algorithms: New models to estimate 10-year risk of cardiovascular disease in Europe. Eur. Heart J..

[15] Zdrojewski T., Jankowski P., Bandosz P., Bartuś S., Chwojnicki K., Drygas W., Gaciong Z., Hoffman P., Kalarus Z., Kaźmierczak J. (2015). A new version of cardiovascular risk assessment system and risk charts calibrated for Polish population. Kardiol. Pol..

[16] Csenteri O., Jancsó Z., Szöllösi G.J., Andréka P., Vajer P. (2022). Differences of cardiovascular risk assessment in clinical practice using SCORE and SCORE2. Open Heart.

[17] Boskovic N., Giga V., Djordjevic-Dikic A., Beleslin B., Stojkovic S., Nedeljkovic I., Aleksandric S., Tesic M., Dedic S., Burazor I. (2022). Comparison of SCORE and SCORE 2 risk prediction tools in contemporary very high risk european population. Eur. Heart J..

[18] Rzepka-Cholasinska A., Kasprzak M., Michalski P., Pietrzykowski Ł., Grzelakowska K., Kubica A. (2022). Cardiovascular risk assessment based on SCORE and SCORE2. Med. Res. J..

[19] Selmer R., Lindman A.S., Tverdal A., Pedersen J.I., Njølstad I., Veierød M.B. (2008). Modell for estimering av kardiovaskulaer risiko i Norge. Tidsskr. Laegeforen.

[20] Svinin G.E., Kutsenko V.A., Shalnova S.A., Yarovaya E.B., Imaeva A.E., Balanova Y.A., Kapustina A.V., Muromtseva G.A., Drapkina O.M. (2024). Validation of SCORE2 on a sample from the Russian population and adaptation for the very high cardiovascular disease risk region. PLoS ONE.

[21] Vincent S.R., Schlenk M.A., Horan K.A., Moore B.A. (2024). Incidences and trends of cardiovascular determinants and diagnoses in active duty service members. J. Mil. Veterans Health.

[22] Pavlík V., Šafka V., Pravdová L., Urban M., Lašák P., Tuček M. (2020). Comparison of selected risk factors in cardiovascular diseases in two different populations of the Czech Republic. Cent. Eur. J. Public Health.

[23] Zimmerman F.H. (2012). Cardiovascular Disease and Risk Factors in Law Enforcement Personnel: A Comprehensive Review. Cardiol. Rev..

[24] Gendron P., Lajoie C., Laurencelle L., Trudeau F. (2019). Cardiovascular health profile among Québec male and female police officers. Arch. Environ. Occup. Health.

[25] Zawadzka M., Marszałkowska-Jakubik J., Ejchman-Pac E., Pająk-Tarnacka B., Szymański P. (2025). Assessing Cardiovascular Risk Among Polish Soldiers: Insights Using the POL SCORE Tool. J. Clin. Med..

[26] Vaicaitiene R., Cerniauskiene L.R., Luksiene D.I., Margeviciene L. (2006). Hypercholesterolemia and Smoking Habits of Lithuanian Military Personnel. Mil. Med..

[27] TVN24 (2011). MON do Żołnierzy: Rzućcie Palenie. https://tvn24.pl/polska/mon-do-zolnierzy-rzuccie-palenie-ra188270-ls3537014.

[28] INFOR (2024). W Obiektach MON Będzie Można Używać Wyrobów Tytoniowych Tylko w Palarniach. W Pojazdach Trzeba Będzie Zapytać o Zgodę Pasażerów. https://www.infor.pl/prawo/sluzby-mundurowe/wojsko/6573853,w-obiektach-mon-bedzie-mozna-uzywac-wyrobow-tytoniowych-tylko-w-palarn.html.

[29] Webber B.J., Bornstein D.B., Kiel M.A., Wilkins R.C., Bryant C.X. (2024). Physical Activity and the Health of a Nation: A National Challenge and Collective Response. Am. J. Lifestyle Med..

[30] Santos A.R.D., Ihlenfeld M.F.K., Olandoski M., Barreto F.C. (2022). Comparative analysis of the health status of military police officers and firefighters: A cross-sectional study in the State of Paraná, Brazil. BMJ Open.

[31] (2023). World Obesity Atlas 2023. 17 World Obesity Federation. https://worldobesity.org/resources/resource-library/world-obesity-atlas-2023.

[32] American Security Project (2023). Combating Military Obesity: Understanding the Problem and Developing Solutions. https://www.americansecurityproject.org/wp-content/uploads/2023/10/Ref-0286-Combating-Military-Obesity.pdf.

[33] Rosellini A.J., Stein M.B., Benedek D.M., Bliese P.D., Chiu W.T., Hwang I., Monahan J., Nock M.K., Petukhova M.V., Sampson N.A. (2017). Using self-report surveys at the beginning of service to develop multi-outcome risk models for new soldiers in the U.S. Army. Psychol. Med..

[34] McBride D., Samaranayaka A., Richardson A., Gardner D., Shepherd D., Wyeth E., de Graaf B., Derrett S. (2022). Factors associated with self-reported health among New Zealand military veterans: A cross-sectional study. BMJ Open.

