# Supplementary material for: SCORE2 Outperforms Pol-SCORE in Detecting Increased Cardiovascular Risk
Source: Pathophysiology. 2025 Sep 9;32(3):45. doi: 10.3390/pathophysiology32030045 (PMC12452680; doi:10.3390/pathophysiology32030045)
Supplement: Supplementary file 1 [file pathophysiology-32-00045-s001.zip › pathophysiology-3795651-supplementary.pdf]

ANKIETA

Ankieta oceniająca stan wiedzy żołnierza/pracownika wojska na temat chorób sercowo-naczyniowych oraz czynników ryzyka ich występowania

Szanowni Państwo, zwracamy się z prośbą o udzielenie odpowiedzi na pytania zawarte w poniższej ankiecie. Badanie ankietowe przeprowadzane jest przez Wojskowy Instytut Higieny i Epidemiologii im. gen. Karola Kaczkowskiego w Warszawie w ramach realizacji projektu NPZ 2021-2025 MON „Narażenie na czynniki środowiskowe (chemiczne, biologiczne i fizyczne) w służbie żołnierzy Wojsk Lądowych, Wojsk Specjalnych i Marynarki Wojennej oraz w powiązanych środowiskach pracy pracowników wojska".

Ankieta ma na celu pozyskanie informacji dotyczących Państwa wiedzy na temat chorób sercowo-naczyniowych oraz czynników ryzyka tych chorób. Dane te posłużą zdefiniowaniu ewentualnych potrzeb edukacyjnych dedykowanych dla żołnierzy i pracowników wojska w zakresie w/w tematyki. Celem projektu jest podniesienie świadomości żołnierzy i pracowników wojska na temat chorób sercowo-naczyniowych.

Uwaga! Ankieta jest anonimowa (prosimy nie podawać danych osobowych oraz danych dotyczących miejsca zatrudnienia). Udział w badaniu ankietowym jest dobrowolny.

Dziękujemy za poświęcony czas

I. PYTANIA DOTYCZĄCE OGÓLNYCH DANYCH DOTYCZĄCYCH OSOBY ANKIETOWANEJ

Płeć

a) męska

b) żeńska

Wiek

Rok urodzenia: ………………………….

Miejsce zamieszkania

a) wieś

b) miasto poniżej 50 tyś. mieszkańców

c) miasto od 50 tyś. do 200 tyś. mieszkańców

d) miasto powyżej 200 tyś. mieszkańców

Wykształcenie

a) podstawowe

b) zawodowe

c) średnie

d) wyższe

Stan cywilny

a) panna/kawaler

b) zamężna/żonaty

c) wdowa/wdowiec

d) rozwiedziona/rozwiedziony

Czy jest Pani/Pan zatrudniona/y jako:

a) żołnierz zawodowy

b) inny pracownik wojska

Ile lat wynosi Pani/Pana staż pracy/służby w jednostce podległej MON:

……………………………………

Proszę wskazać Pani/Pana korpus zawodowy służby wojskowej:

a) szeregowy zawodowy

b) podoficer

c) oficer młodszy

d) oficer starszy

e) generał/admirał

f) nie mam stopnia wojskowego

II. PYTANIA DOTYCZĄCE OCENY WIEDZY NA TEMAT CHORÓB SERCOWO-NACZYNIOWYCH

1. **Czy uważa Pan/Pan, że choroby sercowo-naczyniowe są istotne z punktu widzenia zdrowia publicznego?**
a) tak
b) nie
c) nie wiem
2. **Spośród poniższego wykazu chorób – proszę wskazać te, które należą do schorzeń sercowo-naczyniowych:** **/możliwość wskazania więcej niż jednej odp./**
a) choroba niedokrwienna serca
b) nadciśnienie tętnicze
c) miażdżyca
d) zawał mięśnia sercowego
e) arytmie serca
f) choroby naczyń mózgowych
g) choroby naczyń obwodowych
3. **Najbardziej charakterystycznym objawem choroby niedokrwiennej serca jest:**
a) ból w klatce piersiowej
b) duszność
c) zaburzenia widzenia
d) uczucie kołatania serca
e) wzrost ciśnienia tętniczego krwi
f) omdlenia
g) ból brzucha
h) bóle i zawroty głowy
4. **Prawidłowa wartość ciśnienia u zdrowej osoby powinna wynosić:**
a) poniżej 120/80 mmHg
b) poniżej 140/90 mmHg
c) poniżej 160/100 mmHg
5. **Nadciśnienie tętnicze to choroba, w której obserwuje się:**
a) spadek ciśnienia tętniczego krwi
b) wzrost ciśnienia tętniczego krwi
c) prawidłowe ciśnienie tętnicze krwi
6. **Miażdżyca to choroba związana z:** **/możliwość wskazania więcej niż jednej odp./**
a) nieprawidłowym poziomem lipidów (tłuszczów) we krwi
b) prawidłowym poziomem lipidów (tłuszczów) we krwi
c) związana jest z odkładaniem blaszek miażdżycowych w ścianach naczyń krwionośnych
d) związana jest z nieprawidłową gospodarką węglowodanową
7. **Zawał mięśnia sercowego to inaczej:**
a) martwica mięśnia sercowego
b) miażdżyca
c) nadciśnienie tętnicze
d) wszystkie odpowiedzi prawidłowe
8. **Do chorób naczyń mózgowych należą:** **/możliwość wskazania więcej niż jednej odp./**
a) udar mózgu
b) tętniak mózgu
c) krwotok podpajęczynówkowy
d) ostra encefalopatia nadciśnieniowa
e) przewlekła miażdżyca naczyń mózgu
f) stłuczenie i wstrząśnienie mózgu
9. **Do chorób naczyń kończyn dolnych należy:** **/możliwość wskazania więcej niż jednej odp./**
a) chromanie
b) gangrena
c) płaskostopie
d) miażdżyca tętnic
e) zapalenia naczyń krwionośnych
f) niewydolność żylna
g) zakrzepica żylna
h) żylaki kończyn dolnych
i) tętniak

III. PYTANIA DOTYCZĄCE WIEDZY NA TEMAT CZYNNIKÓW RYZYKA CHORÓB SERCOWO-NACZYNIOWYCH

1. Co to są czynniki ryzyka chorób sercowo-naczyniowych?

a) czynniki zmniejszające ryzyko występowania chorób sercowo-naczyniowych
b) czynniki zwiększające ryzyko występowania chorób sercowo-naczyniowych
c) czynniki nie wpływające na ryzyko występowania chorób sercowo-naczyniowych
d) żadne z powyższych

2. Do czynników ryzyka chorób sercowo-naczyniowych należą: /możliwość wskazania więcej niż jednej odp./

a) wiek
b) płeć
c) występowanie chorób sercowo-naczyniowych w rodzinie
d) błędy dietetyczne
e) mała aktywność fizyczna
f) palenie tytoniu
g) picie alkoholu
h) nadwaga
i) otyłość
j) zwiększony poziom cholesterolu we krwi
k) cukrzyca typu 2
l) stres

3. Nieprawidłowe żywienie jako czynnik ryzyka choroby sercowo-naczyniowej polega na: /możliwość wskazania więcej niż jednej odp./

a) nieregularnym spożywaniu posiłków
b) zwiększonej podaży w diecie tłuszczów zwierzęcych
c) zmniejszonej podaży w diecie tłuszczów zwierzęcych
d) zwiększonej podaży produktów roślinnych

4. Mała aktywność fizyczna może być przyczyną: /możliwość wskazania więcej niż jednej odp./

a) otyłości
b) nadciśnienia tętniczego
c) choroby niedokrwiennej serca
d) zawału serca

5. tzw. „zły cholesterol" to inaczej:

a) cholesterol całkowity
b) LDL
c) HDL
d) trójglicerydy
e) wszystkie wymienione

6. Zwiększony poziom cholesterolu we krwi może być przyczyną: /możliwość wskazania więcej niż jednej odp./

a) nadciśnienia tętniczego
b) zawału serca
c) odkładania blaszek miażdżycowych w ścianach tętnic
d) udaru mózgu

7. Występowanie otyłości lub nadwagi może być przyczyną: /możliwość wskazania więcej niż jednej odp./

a) udaru mózgu
b) miażdżycy tętnic
c) nadciśnienia tętniczego

8. Palenie tytoniu: /możliwość wskazania więcej niż jednej odp./

a) nie ma wpływu na występowanie chorób sercowo-naczyniowych
b) zwiększa ryzyko występowania miażdżycy
c) zwiększa ryzyko występowania zawału serca
d) zwiększa ryzyko udaru mózgu

9. Czynnikiem ryzyka występowania chorób sercowo-naczyniowych jest:

a) wiek powyżej 30 roku życia
b) wiek powyżej 40 roku życia
c) wiek powyżej 50 roku życia
d) wiek nie ma wpływu na występowanie chorób sercowo-naczyniowych

10. Czynnikiem ryzyka występowania chorób sercowo-naczyniowych jest:

a) płeć męska
b) płeć żeńska
c) płeć nie ma znaczenia jako czynnik ryzyka chorób sercowo-naczyniowych

IV. PYTANIA DOTYCZĄCE WIEDZY NA TEMAT PODSTAWOWEJ DIAGNOSTYKI I BADAŃ PRZESIEWOWYCH W CHOROBACH SERCOWO-NACZYNIOWYCH

1. Specjalista zajmujący się diagnostyką i leczeniem chorób serca to:

a) neurolog
b) gastrolog
c) kardiolog
d) urolog

2. Do badań diagnostycznych chorób sercowo-naczyniowych należą: /możliwość wskazania więcej niż jednej odp./

a) gastroskopia
b) EKG
c) koronarografia
d) badanie Dopplera
e) mammografia

3. Oznaczenie poziomu cholesterolu we krwi jest:

a) nieistotnym badaniem przy podejrzeniu miażdżycy
b) podstawowym badaniem przy podejrzeniu miażdżycy
c) dedykowane wyłącznie dla osób po zawale serca
d) badaniem rekomendowanym tylko dla kobiet

V. PYTANIA DOTYCZĄCE STANU ZDROWIA ANKIETOWANEGO W ASPEKCIE WYSTĘPOWANIA CHORÓB SERCOWO-NACZYNIOWYCH

1. Czy w Pani/Pana najbliżej rodzinie występują/-owały choroby sercowo-naczyniowe?

a) tak
b) nie
c) nie wiem

2. Czy choruje Pani/Pan na choroby sercowo-naczyniowe:

a) tak
b) nie
c) nie wiem

3. Czy występują u Pani/Pana następujące objawy: /możliwość wskazania więcej niż jednej odp./

a) bóle w klatce piersiowej
b) duszności
c) zawroty głowy
d) uczucie kołatania serca
e) bóle kończyn dolnych
f) bóle głowy
g) podwyższone wartości ciśnienia tętniczego krwi

4. Czy choruje Pani/pan na następujące choroby: /możliwość wskazania więcej niż jednej odp./

a) nadciśnienie tętnicze
b) miażdżycę
c) chorobę niedokrwienną serca
d) choroby naczyń mózgowych
e) nie wiem
f) nie choruję

5. Jak Pani/Pan ocenia swój stan zdrowia w aspekcie występowania chorób sercowo-naczyniowych?

a) bardzo dobrze
b) dobrze
c) dostatecznie
d) źle
e) nie wiem

6. Jak często wykonuje Pani/Pan badania kontrolne układu sercowo-naczyniowego, takie jak EKG i oznaczenie poziomu cholesterolu we krwi, pomiar ciśnienia tętniczego krwi:

a) w ogóle nie wykonuję
b) raz na 5 lat
c) raz w roku
d) częściej niż raz w roku
e) badam się wyłącznie wtedy, kiedy mam objawy
f) badam się tylko w ramach badań okresowych u lekarza medycyny pracy

7. Czy według Pani/Pana występują u Pani/Pana następujące czynniki ryzyka chorób sercowo-naczyniowych: /możliwość zaznaczenia więcej niż jednej odp./

a) nadwaga
b) otyłość
c) niewłaściwa dieta
d) podwyższony poziom cholesterolu we krwi
e) cukrzyca typu 2
f) mała aktywność fizyczna
g) palenie tytoniu
h) picie alkoholu
i) stres
j) występowanie chorób sercowo-naczyniowych w rodzinie
k) żadne z powyższych

8. Czy potrafi Pani/Pan oznaczyć wskaźnik BMI (Body Mass Index)?

a) tak
b) nie

9. Czy Pani/Pan paliła/- w przeszłości?

a) tak
b) nie
c) Czy Pani/Pan pali?
d) tak, papierosy
e) tak, e-papierosy
f) tak, fajkę
g) nie

10.Jak długo Pan/i pali?

a) nie palę
b) do 5 lat
c) od 5 do 10 lat
d) od 10 do 15 lat
e) od 15 do 20 lat
f) powyżej 20 lat

11.Ile sztuk dziennie Pan/i pali?

a) do 5 sztuk
b) 6-10 sztuk
c) 11-20 sztuk
d) powyżej 20 sztuk

12.Jak często spożywa Pani/Pan alkohol?

a) codziennie
b) dwa-trzy razy w tygodniu
c) raz w tygodniu
d) rzadziej
e) w ogóle nie piję alkoholu

13.Ile razy w tygodniu Pani/Pan wykonuje ćwiczenia fizyczne trwające powyżej 30 minut?

a) codziennie
b) 5-6 razy w tygodniu
c) 3-4 razy w tygodniu
d) 1-2 razy w tygodniu
e) rzadziej niż raz w tygodniu

VI. PYTANIA DOTYCZĄCE ŚRODOWISKA SŁUŻBY/PRACY W ASPEKCIE WYSTĘPOWANIA CZYNNIKÓW RYZYKA CHORÓB SERCOWO-NACZYNIOWYCH

1. Czy w Pani/Pana środowisku służby/pracy występują czynniki ryzyka sprzyjające rozwojowi chorób sercowo-naczyniowych? /możliwość zaznaczenia więcej niż jednej odp./

a) mała aktywność fizyczna
b) stres
c) nieprawidłowe żywienie
d) inne
e) nie występują

2. W jakim stopniu Pani/Pana praca/służba jest czynnikiem stresu?

a) znacznym
b) średnim
c) małym
d) praca/służba nie jest dla mnie źródłem stresu
e) nie wiem

VII. PYTANIA DOTYCZĄCE OCENY WŁASNEJ WIEDZY NA TEMAT CZYNNIKÓW RYZYKA CHORÓB SERCOWO-NACZYNIOWYCH I CHORÓB SERCOWO-NACZYNIOWYCH

1. Jak Pani/Pan ocenia własną wiedzę na temat rozpoznawania i diagnostyki chorób sercowo- naczyniowych?

a) bardzo dobrze
b) dobrze
c) dostatecznie
d) poniżej dostatecznie
e) nie wiem

2. Jak ocenia Pani/Pan własną wiedzę na temat czynników ryzyka chorób sercowo-naczyniowych?

a) bardzo dobrze
b) dobrze
c) dostatecznie
d) poniżej dostatecznie
e) nie wiem

3. Czy potrafi Pani/Pan wskazać metody profilaktyki chorób sercowo-naczyniowych, które można stosować w codziennym życiu?

a) tak
b) nie
c) nie wiem

4. Do metod profilaktycznych w chorobach sercowo-naczyniowych należą: /możliwość zaznaczenia więcej niż jednej odp./

a) dieta bogatotłuszczowa
b) dieta niskotłuszczowa
c) małe spożycie soli
d) aktywny tryb życia
e) redukcja masy ciała
f) rzucenie nałogu palenia papierosów

VIII. PYTANIA DOTYCZĄCE EDUKACJI W ZAKRESIE CZYNNIKÓW RYZYKA CHORÓB SERCOWO-NACZYNIOWYCH I CHORÓB SERCOWO-NACZYNIOWYCH

1. Skąd pozyskuje Pani/Pan wiedzę na tematy zdrowotne, w tym o chorobach sercowo-naczyniowych? /możliwość zaznaczenia więcej niż jednej odp./

a) z internetu
b) z gazet i książek
c) z publikacji naukowych
d) od znajomych
e) z telewizji
f) z innych źródeł
g) nie interesuję się ww. tematem

2. Czy chciałby Pan/Pani poszerzyć wiedzę na temat czynników ryzyka chorób sercowo-naczyniowych i chorób sercowo-naczyniowych?

a) tak
b) nie
c) nie wiem

3. Czy uważa Pani/Pan, że szkolenia dotyczące zapobiegania, wczesnego wykrywania, leczenia chorób sercowo-naczyniowych powinny być cyklicznie realizowane w Siłach Zbrojnych RP?

a) tak
b) nie
c) nie wiem
